# DenseLes: slice-wise dense network for multiple sclerosis lesion segmentation and classification

**DOI:** 10.3389/fneur.2026.1704317

**Published:** 2026-02-26

**Authors:** Melinda Katona, Bence Bozsik, Péter Bodnár, Krisztián Kocsis, Eszter Tóth, Nikoletta Szabó, András Király, Péter Faragó, László G. Nyúl, Dániel Veréb, Zsigmond Tamás Kincses

**Affiliations:** 1 Department of Radiology, University of Szeged, Szeged, Hungary; 2 Department of Image Processing and Computer Graphics, University of Szeged, Szeged, Hungary; 3 Department of Neurology, University of Szeged, Szeged, Hungary

**Keywords:** brain extraction, brain MRI, convolutional neural networks (CNN), lesion segmentation, multiple sclerosis

## Abstract

Accurate and reliable segmentation of multiple sclerosis (MS) lesions from magnetic resonance imaging (MRI) is essential for diagnosis and monitoring disease progression. Therefore, a robust and efficient automated approach can rapidly provide information about the patient. Here, a convolutional neural network-based method is proposed to segment lesions from FLAIR images. The DenseLessystem includes two stages: pre-processing of image data (brain extraction, standardization), then segmentation of MS lesions using an end-to-end slice-wise dense network. We also identified the segmented lesions in specific locations [periventricular, (juxta)cortical, infratentorial, and spinal]. DenseLesis evaluated and compared to other methods on our assembled data and the public MSSEG 2016 MS challenge dataset. Our model demonstrates a significant improvement in segmentation quality over previous approaches, achieving an average Dice score of 0.80% on the Szeged MS dataset. On the MSSEG 2016 dataset, our method achieved Dice scores ranging from 0.32% to 0.73%, comparable to those of human raters.

## 1 Introduction

Multiple Sclerosis (MS) is a chronic, progressive disease of the central nervous system in young adults leading to demyelination and axonal loss (1). Conventional T1(T1w)-, T2-weighted (T2w), and Fluid Attenuated Inversion Recovery (FLAIR) MR images play a key role in the diagnosis and follow-up of MS. Macroscopic white matter lesions (WML); which appear as hyperintense circumscribed areas on T2w images are important biomarkers of MS. Although they correspond to various histopathological changes such as edema or demyelination (2) the number and volume of these lesions correlate with the clinical disability and cognitive dysfunction of the patients (3). The radiological diagnosis is based on the identification of WMLs in certain key locations [periventricular, (juxta)cortical, infratentorial, spinal]. Hence, identifying WMLs at various locations and estimating their volume are crucial. However, manually identifying and segmenting WMLs is tedious and time-consuming. The reproducibility of manual lesion identification is modest.

Numerous automated approaches have been proposed to tackle this problem. Automated segmentation of MS lesions is challenging: lesion size and location are highly variable, and lesion boundaries are often poorly defined. Furthermore, images may have low resolution and exhibit imaging artifacts. Several automated methods have been proposed over the past decade. These approaches are usually divided into two categories, supervised and unsupervised. Supervised segmentation algorithms use a priori knowledge from a training dataset. Most of these methods are based on Convolutional Neural Networks. Unsupervised algorithms rely on intensity models of brain tissue. These approaches include thresholding (4, 5), modeling voxel intensities, and identifying outliers (6–8). Despite the availability of various automated methods for MS lesion segmentation, the need for a more reliable approach is evident from the continued organization of annual competitions aimed at maximizing segmentation accuracy.

Recent literature on MS lesion segmentation demonstrates substantial progress beyond standard U-Net architectures, with a focus on robustness and advanced feature extraction. While highly optimized 3D U-Net frameworks like nnU-Net (9) serve as robust baselines, as demonstrated by the FLAMeS model (10), a significant trend is the integration of Transformers to capture global context. For instance, a recent study proposed a hybrid Transformer-CNN framework that explicitly combines local CNN features with a Transformer's ability to model long-range dependencies (11). Beyond architecture, researchers are increasingly adopting multi-scale and multimodal strategies to address biological heterogeneity. In digital pathology, Selcuk et al. (12) introduced a pyramid sampling strategy to capture features at multiple spatial scales, effectively reducing subjectivity in biomarker scoring by analyzing both cellular details and tissue architecture. Similarly, recent advancements in musculoskeletal modeling have employed multimodal frameworks—combining medical imaging with kinematic deep learning—to achieve high-precision, subject-specific tissue analysis (13).

In this study, we introduce a novel, open-source, deep learning-based approach to segment MS lesions, which relies on dense convolutional neural networks (DenseLes). We evaluate the efficacy of the suggested approach on public databases and local data compared to other available lesion segmentation methods [BIANCA (14), FLEXCONN (15), and LST-LPA].

## 2 Materials and methods

### 2.1 Datasets

To validate our approach, we used a publicly available dataset to evaluate and compare the proposed method with existing methods in the literature. A local clinical dataset was used to train and cross-validate the DenseLesarchitecture. We describe the datasets below:

**MSSEG 2016**^1^: The images used for comparison originated from the MICCAI MS segmentation challenge dataset (16). This database comprises scans of 15 MS patients acquired on three various MRI scanners (Siemens Vario, Philips Ingenia, Siemens Area) using 3T and 1.5T magnetic field strengths. For each MS patient, four sequences are provided; however, we used only FLAIR images in our analysis. Furthermore, seven expert lesion delineations are available, and a consensus ground-truth segmentation was constructed for evaluation.

**Szeged MS dataset**: The dataset comprises images from 91 subjects acquired on a 3T GE Discovery 750 w MR scanner. For lesion segmentation, CUBE T2 FLAIR images were used with the following parameters: TE: 135 ms; TR: 6700 ms; TI: 1827 ms; matrix: 256 × 224; FOV: 25 × 22.5 cm; slice thickness: 1.4 mm. An expert rater, BB, manually outlined lesions on 3D FLAIR images under the supervision of ZTK, who has more than 10 years of experience in neuroradiology.

### 2.2 Pre-processing

We applied intensity standardization (17) to transform voxel intensities to a standard grayscale range. To aid lesion segmentation, brain extraction was performed with DeConvBET, developed by our group (Katona et al., submitted). The image volumes were resampled to 1 mm × 1 mm × 1 mm and registered to standard space using the T1-weighted FSL MNI template (MNI152_T1).

#### 2.2.1 DenseLes implementation details

The heterogeneity of MS lesion locations and sizes poses a challenge for detection and segmentation in MR images. In this study, the architecture is based on the U-Net (18) with modifications. Three 2D densely connected networks were trained on the clinical dataset. In a dense block, each layer receives inputs from all preceding layers and passes its feature maps to all subsequent layers. Volumes were extracted into 2D slices from all three orthogonal views of the brain for training.

The core of our model is a custom densenet_block. Its core idea is feature reuse. Unlike a standard network, where each layer receives input from the immediately preceding layer, each layer in a Dense Block receives feature maps from all preceding layers within that block. The encoder (contracting) path includes an initial 2D convolutional layer and two levels of feature extraction. The bottleneck layer, which builds on features extracted by the encoder, consists of a single densenet_block. The decoder (expansive) path symmetrically reconstructs the image resolution. At each decoder stage, the up-sampled feature maps are concatenated with high-resolution feature maps from the encoder via long-range skip connections. Our custom densenet_block creates a wide, feature-rich representation by concatenating the outputs of four internal convolutional sub-blocks.

**Figure 1:**
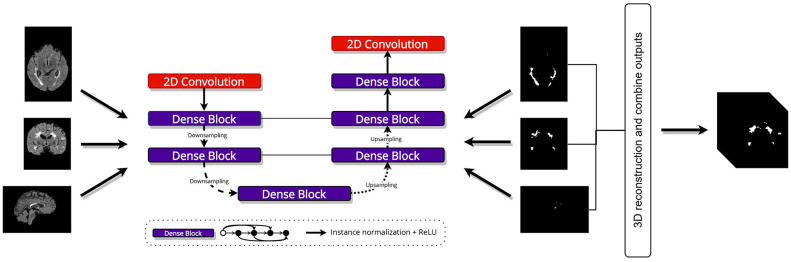
A general overview of the proposed lesion segmentation network. Our approach produces a 3D volumetric binary map by combining the 2D outputs of the networks. The input image slices are obtained from each plane orientation (axial, coronal, and sagittal), trained individually, and then a majority vote is applied to obtain the final segmentation result. Input shapes are the following **(top to bottom)**: (160, 256), (160, 176), and (256, 176).

The images in different directions have different sizes. Thus, we trained three models according to orientations, and the output probability maps were combined using majority voting after a 3D reconstruction to obtain a single binary lesion volume as illustrated in Figure 1.

Data imbalance is a common issue in medical image segmentation, including lesion detection. Only a limited number of annotated images are available for development. Additionally, only a small number of voxels are lesions, which reduces the number of positive training samples. The number of non-lesion voxels is considerably larger than the number of lesion points. The learning process may converge to a local minimum of a suboptimal loss function when labels are not balanced. To address this issue, we used Tversky-loss defined as the following:


$$\begin{array}{l} {\text{L}}_{t}=1-\frac{\sum_{i=1}^{NM}y_{0i}\cdot {ŷ}_{0i}+\epsilon }{\sum_{i=1}^{NM}y_{0i}\cdot {ŷ}_{0i}+\alpha \sum_{i=1}^{NM}y_{0p}\cdot {ŷ}_{1i}+\beta \sum_{i=1}^{NM}y_{1i}\cdot {ŷ}_{0i}+\epsilon } \end{array}$$ (1)

where *y*_0*i*_, ŷ_0*i*_ is the probability of voxel *i* be a lesion and *y*_1*i*_, ŷ_1*i*_ is the probability of *i* be a non-lesion. Hyperparameters α and β control the magnitude of penalties for false positives and negatives, respectively.

We used α = 0.3, β = 0.7. The rationale for choosing β > α was to place a higher penalty on false negatives (missed lesions).

The network training was performed using RMSprop for optimization with the momentum of 0.95 and the initial learning rate of 1*e*^−3^. The trained networks were expected to receive a 2D slice as input and to predict a 2D probability lesion map. All 2D convolutional layers use 3 × 3 kernel size and He normal (19) to initialize network weights. The dataset was divided into training and validation subsets 10 times. We applied the early stopping technique as regularization to mitigate overfitting by monitoring performance on the validation set using the Dice coefficient (refer to Section 2.3 for details). Finally, to reconstruct the 3D volume, concatenated 2D outputs using the original input data were employed for each slice orientation. We combined the 3D probability maps using majority voting to produce a single lesion segmentation volume as the output.

To manage the training process and prevent overfitting, we employed an early stopping mechanism. This technique monitors the model's performance on a separate validation dataset after each epoch. We selected the Tversky loss as the primary metric to monitor because it is well-suited to the severe class imbalance inherent in MS lesion segmentation. Training was configured to automatically terminate if this validation loss failed to improve for a pre-defined number of consecutive epochs.

#### 2.2.2 Post-processing

False lesions are most common in the choroid plexus and pineal gland, as more hyperintensive areas on FLAIR images, similar to lesions, may also occur here. Importantly, these structures appear on the T1-weighted images as iso- or hypointense to the cortex. We applied a post-processing step that included intensity-based tissue-type segmentation of the 3DT1 FSPGR images, followed by registration and masking of the FLAIR images. FSL FAST (20) tissue-type segmentation was carried out on the brain extracted, bias-field corrected 3D T1 weighted images. T1-weighted images were registered to FLAIR by 6DOF linear registration and trilinear interpolation by FLIRT (ref: FLIRT). CSF partial-volume images, thresholded at 0.5 and binarized, were used to mask FLAIR images and remove the aforementioned structures.

#### 2.2.3 Lesion classification

To identify periventricular lesions, the CSF partial volume images created from the 3DT1 images using FAST (20) were transformed into FLAIR space, thresholded at 0.5 probability, and binarized. The mask was dilated with a 7 × 7 spherical element, and the original image was then subtracted from the lesion mask. (Juxta)Cortical lesions were identified similarly using the cortical gray matter partial volume images. To localize infratentorial lesions, we used a brain stem and cerebellum template from the Oxford brain atlas included in fsleyes,^2^ which was transformed into the FLAIR space of the current patient to determine overlapping regions with the lesion mask. While calculating lesion volume is straightforward from segmented lesions, determining the number of lesions can be difficult when lesions are confluent. Lesions may be located close to each other or even become confluent, especially in the case of periventricular lesions. Therefore, these regions may contain connected components that can lead to errors in lesion count estimation. We used FSL's Gaussian Random Field-based *cluster* method. The original image volume is also required in addition to the binary lesion image. The local maxima identified by the analysis were used to count the lesions in the above-mentioned locations.

### 2.3 Goodness of segmentation

We applied widely used and relevant metrics to evaluate and compare the methods.

The Dice similarity coefficient measures the overlap between segmentation results and annotations. Mathematically, it is expressed as


$$\begin{array}{l} \text{Dice}\left(X,Y\right)=2\cdot \frac{\left|X\cap Y\right|}{\left|X|\cup |Y\right|} \end{array}$$ (2)

where X denotes the manual mask, and Y is also a binary vector representing the predicted mask.

Furthermore, for better comparison, we also used object-wise metrics. The object-level Dice score given as:


$$\begin{array}{l} {\text{Dice}}_{obj}\left(X,Y\right)=\frac{1}{2}\cdot \left[\overset{n_{Y}}{\underset{i=1}{\sum }}w_{i}D\left(X_{i},Y_{i}\right)+\overset{n_{X}}{\underset{j=1}{\sum }}{\tilde{w}}_{j}D\left({\tilde{X}}_{j},{Ỹ}_{j}\right)\right] \end{array}$$ (3)

where $X,\tilde{X}$ denote reference mask, *Y*, Ỹ are the predicted mask, *i* and *j* refer to the actual object. This metric compares ground truth objects to the predicted, and vice versa. The weight of each object is determined as follows: $w_{i}=|Y_{i}|/\overset{n_{Y}}{\underset{j=1}{\sum }}|Y_{j}|$ and ${\tilde{w}}_{j}=|\tilde{X_{i}}|/\overset{n_{X}}{\underset{j=1}{\sum }}|\tilde{X_{j}}|$.

Positive predictive value, also called precision, is defined as the ratio of true positives to the sum of the false positives:


$$\begin{array}{l} \text{PPV}=\text{TP}/\left(\text{TP}+\text{FP}\right) \end{array}$$ (4)

where FN is the lesion regions wrongly classified as non-lesion regions, FP denotes the non-lesion regions detected as lesions, and TP is the accurately classified lesion regions.

True positive rate or Recall gives the fraction of positive objects that are correctly identified: TPR = TP/(TP + FN), while False positive rate (FPR) = FP/(FP + TN), where TN is the number of true negatives. We considered the object-level true positive rate (OTPR) and the false positive rate (OFPR), and defined lesions as groups of 26-connected voxels.

The average symmetric surface distance (ASSD) is the average of all distances between the boundary of the segmented and the mask region.


$$\begin{array}{l} \text{ASSD}\left(X,Y\right)=\frac{1}{\left|X|+|Y\right|}\left(\underset{x\in X}{\sum }D_{Y}\left(x\right)+\underset{y\in Y}{\sum }D_{X}\left(y\right)\right) \end{array}$$ (5)

where *D* denotes the Euclidean distance metric. ASSD is measured in millimeters.

The size of the MS lesions can vary considerably. Hence, we analyzed the goodness of segmentation on the MICCAI 2016 dataset according to lesion size in three groups: small lesions: <5 ml, medium-sized lesions: 5-15 ml, and large lesions >15.

## 3 Results

To ensure a fair and unbiased benchmark, we established a single, unified preprocessing pipeline for all experiments. For both our in-house data and the MSSEG 2016 data, all images were first co-registered to the FLAIR sequence and standardized. Each image was then resampled to a standard voxel size and spatially aligned to a common template.

The same set of preprocessed images was used as input to our proposed DenseLes model and to all baseline methods (BIANCA, FLEXCONN, and LST-LPA). While the internal algorithmic parameters of the baseline methods were kept to their recommended defaults, the key preprocessing and data inputs were held identical. Furthermore, no method-specific post-processing (e.g., small-cluster removal) was applied to the raw outputs of any method. This methodology ensures that all reported performance differences are attributable to the core segmentation algorithms themselves, rather than to a bias in the data preparation workflow.

### 3.1 Szeged MS dataset

As described in Section 2.1, the clinical dataset consists of 91 FLAIR images. Due to the small number of examples, 10x cross-validation was used, with 20% of the data held out as the validation set. In addition to our approach, we also evaluated the results of the BIANCA,^3^ FLEXCONN,^4^ and LST-LPA^5^ methods on each set.

**Figure 2:**
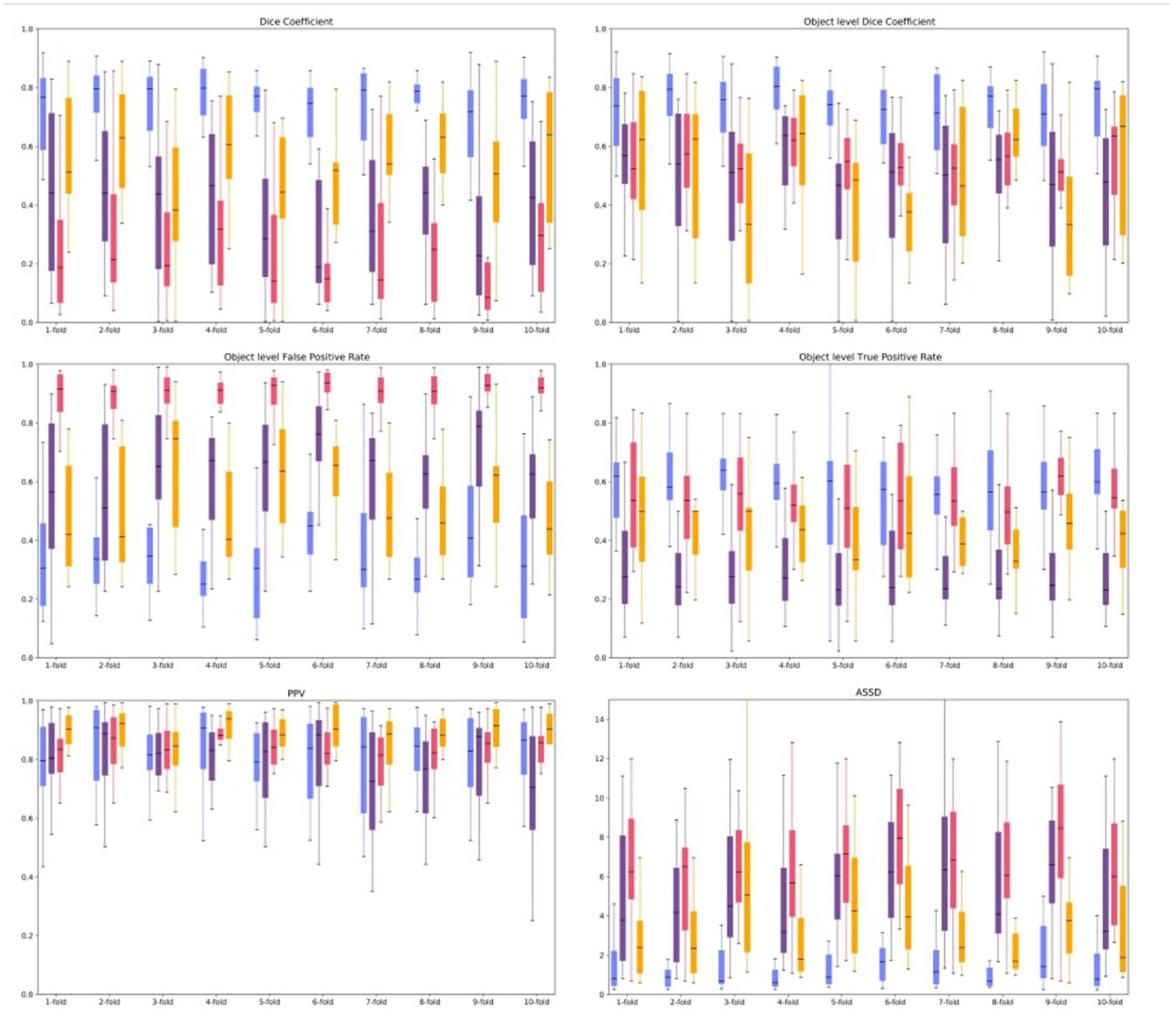
Boxplots of evaluated metrics obtained from the Proposed method (blue box), BIANCA (purple box), FLEXCONN (pink box), and LST-LPA (orange box) on the clinical MS dataset. Similarity metrics are the following: Dice coefficient ↑, Object level dice coefficient ↑, Positive Prediction Value (PPV) ↑, Object level False Positive Rate (OFPR) ↓, Object level True Positive Rate (OTPR) ↑, and Average Symmetric Surface Distance (ASSD) ↓. Black lines denote the median of the measured values.

To rigorously evaluate the generalizability of our model, we employed a 10x cross-validation (CV) scheme. The dataset was partitioned into 10 equal-sized folds using a standard, randomized sampling function. We acknowledge that this randomized approach does not explicitly stratify the data; therefore, the distribution of key clinical characteristics—such as total lesion load or the prevalence of small lesions—may vary between folds. This inherent randomness may contribute to the observed variance in performance metrics across different folds. However, this standard CV methodology is widely adopted to provide an unbiased, averaged estimate of model performance on unseen data. The average of all metrics obtained in the validation set folds of the cross-validation is shown in Figure 2.

**Figure 3:**
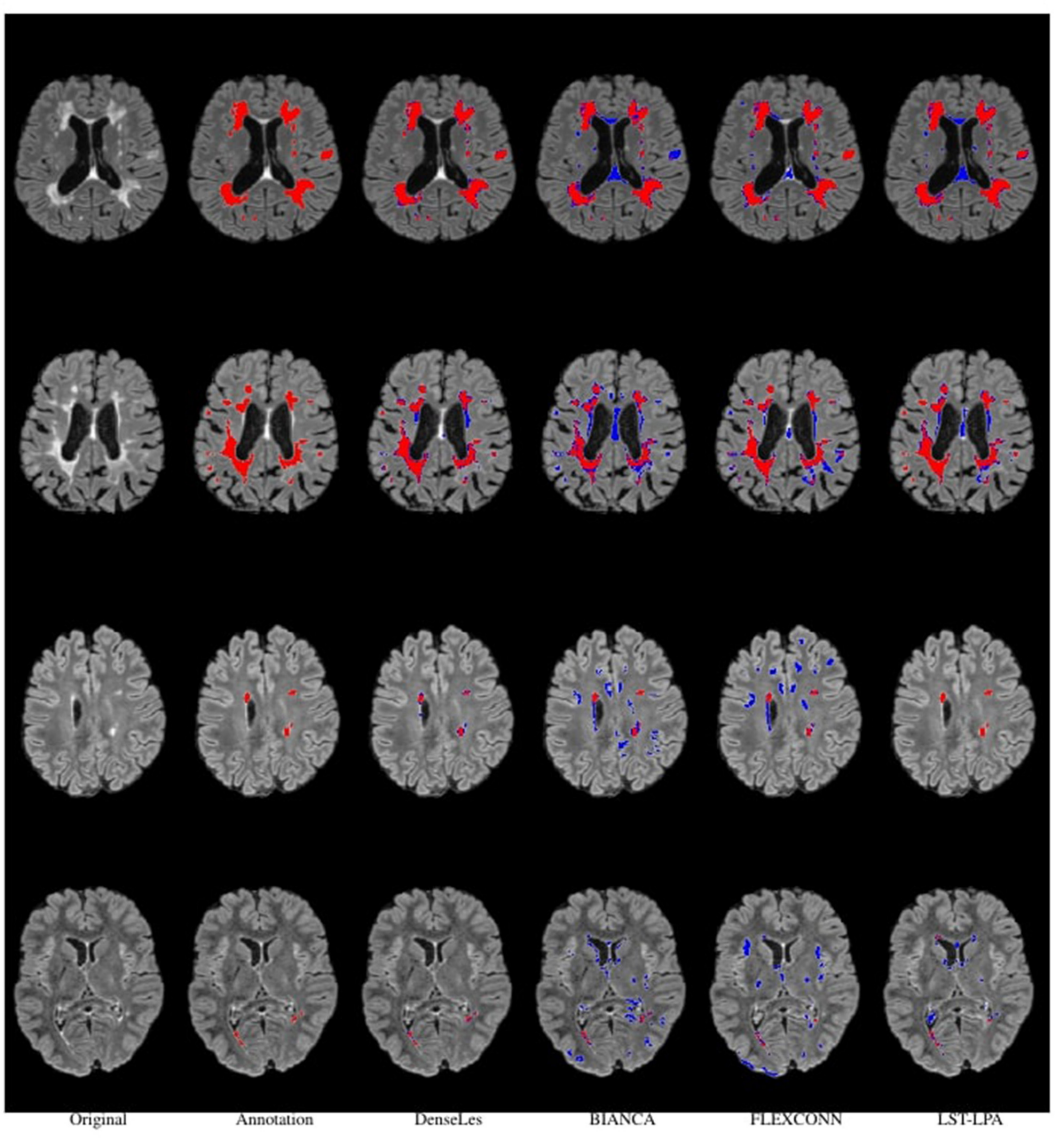
Visual comparison of results for all described methods. The first column represented subjects from the clinical dataset, and the second row contained their annotation masks. Segmented areas are visualized in red. Blue denotes the misclassified voxels.

**Figure 4:**
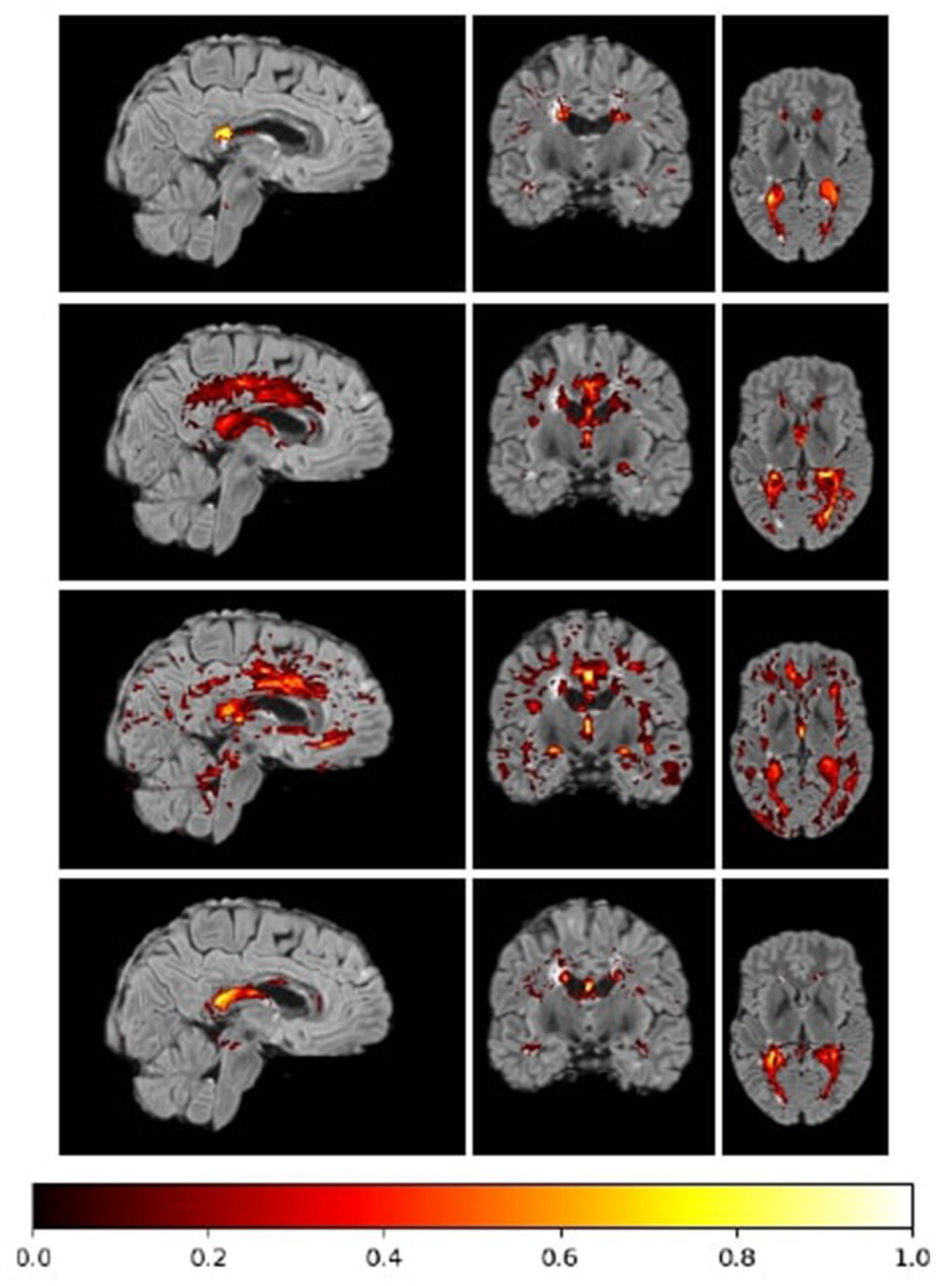
Heatmap of the summarized misclassified voxels in a sample fold normalized to [0, 1] range. More common errors are visualized with brighter colors in the colormap. The maps of the methods from top to bottom: DenseLes, BIANCA, FLEXCONN, and LST-LPA.

DenseLes produced similar results across folds, demonstrating the protocol's reliability in lesion detection. DenseLesachieved a higher value both at the global and object-level Dice score, which quantifies the ratio of overlap, and the standard deviation of the results is smaller than that of the other approaches. DenseLeswas more volatile with respect to PPV. At the object level, we analyzed the ratio of incorrectly to correctly segmented lesions. The OTPR value of FLEXCONN is higher for each fold than BIANCA or LST-LPA, but DenseLesperformed far better than the other approaches. The distance between segmented and annotated objects was also evaluated. We obtained substantially better results for our method in the average symmetric surface distance. The average distance between the annotated mask and the predicted lesions is less than 2 mm. We also present qualitative results in Figures 3, 4. Many false-positive lesions appear in both the literature and the proposed methods. The high number of false positives in the BIANCA and FLEXCONN approaches may explain the lower Dice coefficients.

### 3.2 MSSEG 2016

A direct benchmark of our model against the official MSSEG 2016 challenge leaderboard is not feasible, as the hidden test dataset and the online evaluation platform are no longer publicly available. Therefore, to benchmark our model's performance, we compared our results with those of other recently published deep learning methods that have used the public MSSEG 2016 training dataset for evaluation, typically employing a cross-validation scheme or a custom train-test split.

A common problem with deep learning approaches is that efficiency decreases on unseen data. To assess this issue, the methods were also evaluated on a publicly available dataset, using images from various scanners and vendors. Only the training dataset was available from the MSSEG challenge dataset. Hence, the measures we achieved cannot be compared with the published challenge results. The previously introduced metrics were also estimated to compare the approaches.

We examined the segmentation results separately for different MR vendors. DenseLesachieved good comparable results on various field strengths and vendors (16). Table 1 summarizes results using the MSSEG dataset.

**Table 1:** Experimental results using the MICCAI 2016 challenge on the multiple sclerosis lesion segmentation dataset.

**Scanner**	**Method**	**Dice_*obj*_**	**Dice**	**PPV**	**OFPR**	**OTPR**	**ASSD**
Siemens 3T Verio	DenseLes	0.6869	**0.6377**	**0.7042**	0.7254	0.5290	3.9145
	FLEXCONN	0.4222	0.3971	0.4917	0.9325	**0.7926**	5.9612
	LST-LPA	**0.7044**	0.5665	0.4299	**0.6609**	0.5295	**2.3168**
Siemens Aera 1.5T	DenseLes	**0.5513**	**0.5163**	**0.6432**	0.8149	0.7818	3.9265
	FLEXCONN	0.1988	0.1874	0.3015	0.9120	**0.8048**	7.9887
	LST-LPA	0.1785	0.1545	0.1219	**0.2883**	0.2301	**2.4486**
Philips Ingenia 3T	DenseLes	**0.6858**	**0.4594**	**0.8276**	0.7271	0.4700	5.5625
	FLEXCONN	0.3237	0.2703	0.3758	0.8788	**0.5427**	5.7967
	LST-LPA	0.2628	0.2676	0.2045	**0.2461**	0.2322	**1.3983**

Measured values are expressed in mean (± standard deviation). The best results are highlighted in bold.

The results demonstrated the robust performance and effectiveness of the DenseLes architecture for segmenting complex MS lesions. However, we acknowledge that while these findings are highly promising, the model's generalizability to diverse, unseen multi-center datasets remains a critical next step. The inherent variability in scanner hardware and acquisition protocols across clinical sites presents an ongoing challenge for automated tools. Consequently, further validation in broader, multi-institutional cohorts is intended to establish new benchmarks and standardized datasets, as these become available, ensuring the model's reliability across diverse real-world clinical environments.

It is important to discuss the relationship between model sensitivity and lesion size. Our analysis, consistent with prior literature, confirms that segmentation performance correlates strongly with lesion volume. Larger lesions, which are more easily identified and delineated, are localized effectively by all the methods we examined.

We examined the effect of lesion size on segmentation goodness. We analyzed the MICCAI 2016 dataset, and lesions were split into three groups depending on volumes: low lesion volume (<5 ml, *n* = 205), medium lesion volume (5–15 ml, *n* = 2), and high lesion volume (>15, *n* = 8). The total number (n) of extracted lesion volumes was 215. Our results showed that segmenting smaller lesions poses the greatest challenges for many approaches. Table 2 shows that DenseLes can more efficiently detect lesions for all lesion sizes. The other methods presented perform poorly, which may be because they are designed for segmenting larger-volume objects.

**Table 2:** Mean Dice_*obj*_ detection rate of lesion volumes measured on the MICCAI 2016 dataset.

**Scanner**	**Method**	** <5 ml**	**5 - 15 ml**	**> 15 ml**
Siemens 3T Verio	DenseLes	**0.4778**	**0.4688**	**0.5807**
	FLEXCONN	0.0124	0.0006	0.0374
	LST-LPA	0.0107	0.0000	0.0437
Siemens Aera 1.5T	DenseLes	**0.5873**	**0.7633**	**0.7633**
	FLEXCONN	0.0269	0.0000	0.0573
	LST-LPA	0.0002	0.0000	0.0000
Philips Ingenia 3T	DenseLes	**0.5438**	-	**0.6017**
	FLEXCONN	0.0131	-	0.0408
	LST-LPA	0.0015	-	0.0161

“—” indicates a zero-sample count for that specific category. Bold values indicate the best achieved results.

Conversely, small lesions pose a significant challenge for both automated methods and expert manual raters. This inherent difficulty influenced the curation of our in-house dataset, prompting us to adopt a pragmatic approach by annotating only lesions whose extent could be meaningfully measured.

**Figure 5:**
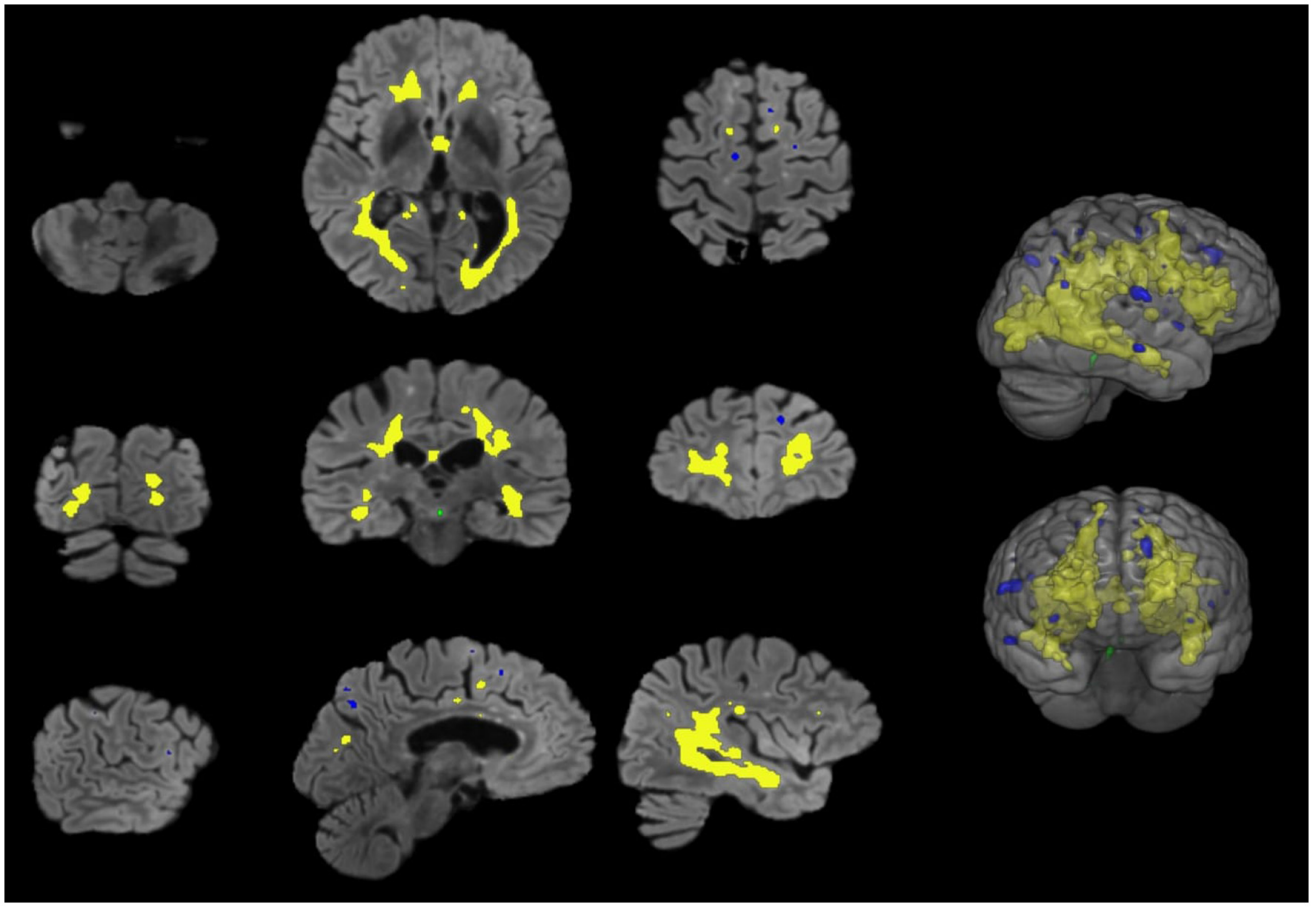
Qualitative assessment of lesion classification approach. The color code for automatically segmented lesions is as follows: infratentorial (green), periventricular (yellow), and juxtacortical (blue).

Figure 5 shows a lesion classification example result.

Following segmentation, our full pipeline includes an automated post-processing step that classifies detected lesions into clinically relevant anatomical regions. Figure 5 provides a qualitative illustration of this classification module's output. Segmented lesions are color-coded according to their assigned anatomical label. This demonstrates our pipeline's end-to-end capability to not only segment lesions but also provide spatial localization.

To evaluate clinical practicality, the computational efficiency of DenseLes was assessed during inference. On a standard workstation equipped with an NVIDIA GeForce RTX 2070 GPU (8GB VRAM) and an Intel Core i7 CPU, the average inference time for a complete 3D MRI volume (approximately 180–200 slices) was approximately 4.2 seconds. This slice-wise processing speed makes the model suitable for near-instantaneous lesion quantification in clinical practice and requires significantly fewer resources than fully volumetric 3D architectures.

## 4 Discussion

A significant challenge in automated multiple sclerosis (MS) lesion segmentation is the lack of robust methods that generalize reliably across data from different acquisition sites and MRI vendors. Although numerous approaches have been proposed, this poor generalizability often limits their clinical utility.

In this study, we introduced DenseLes, a novel deep learning-based method designed to address this challenge. Our approach not only segments white matter lesions from T2-weighted FLAIR images but also localizes them within clinically relevant anatomical regions. The model was developed using a local MS database and was specifically architected to handle common challenges in medical imaging, including severe class imbalance and limited training data.

We demonstrated that the present method outperforms other leading supervised and unsupervised approaches in segmentation accuracy at both the voxel and object (lesion) level. Crucially, we validated that our model maintains its high performance on external, multi-vendor datasets, confirming its robustness and potential for deployment in real-world clinical settings.

The clinical utility of our model is particularly evident in the context of the modern MS treatment paradigm, which aims to achieve “No Evidence of Disease Activity” (NEDA-4). This composite measure is defined by the absence of: (1) clinical relapses, (2) disability progression, (3) new or enlarging T2 lesions, and (4) no significant brain atrophy.

Our FLAIR-based segmentation tool is directly applicable to the third and most challenging of these components: the detection of new or enlarging T2 lesions, a task that is notoriously time-consuming and difficult for human raters, especially when lesions are small. Our tool can be integrated into a longitudinal pipeline by co-registering a patient's baseline and follow-up scans. By segmenting lesions in both images, a subtraction map can be generated to automatically flag newly identified voxels. This highlights a “silent progression” that a manual radiological review might miss, providing objective, reproducible evidence to assess this key NEDA-4 component.

Brain Intensity AbNormality Classification Algorithm (BIANCA) (21) is a supervised, multimodal method for white matter hyperintensity detection based on the kNN algorithm. Guizard et al. (14) demonstrates a rotation-invariant multi-contrast non-local means approach to detect structures that vary in size, shape, and location. Maier et al. (22) suggested an Extra Tree forest framework for voxel-wise classification using simple image features. Logistic regression is also a popular method in lesion classification, where models comprise multiple MRI modalities to estimate voxel-level probabilities of lesion presence (23). Whereas the approaches mentioned above were based on voxel-wise intensities, Roy et al. (24) introduced a patch-based method. Patches from multimodal MR images are matched to patches from the atlas, and lesion memberships are derived from patch similarity weights.

In recent years, methods employing Deep Neural Networks (DNNs) have gained popularity across many research fields. Convolutional Neural Networks (CNN)- based methods have become widely used approaches for addressing medical imaging problems and challenges. Valverde et al. (25) proposed a cascade of two 3D patch-wise convolutional neural networks. The first network provides candidate lesion points to the second, thereby reducing the number of misclassified voxels. Recurrent slice-wise attention network (RSANet) (26) models 3D images as sequences of slices and captures long-range dependencies to utilize contextual information about lesions. Hatamizadeh et al. (27) introduced the Deep Active Lesion Segmentation (DALS) framework that leverages the abilities of CNNs and Active Contour Models (ACM). Denner et al. (28) hypothesized that structural changes of lesions between longitudinal MRI scans are valuable cues for improving detection. Fast Lesion EXtraction using COnvolutional Neural Networks (FLEXCONN) (15) is based on parallel pathways of convolutional filters applied to multiple contrasts. The outputs of the pathways are concatenated, and another set of filters is used on the joined output. Ansari et al. (29) defined a pathway consisting of multiple parallel convolutional filter banks catering to multiple MR modalities and another to produce membership functions for lesions that are thresholded to obtain the binary result. Essa et al. (30) proposed an adaptive neuro-fuzzy inference system (ANFIS) to fuse segmentation results of T2-w and FLAIR sequences generated by a Region-Based Convolutional Neural Network. ACU-Net includes a 3D spatial attention block (31) to enrich spatial details and feature representation of lesions in the decoding stage. Furthermore, a 3D context-guided module was designed for exploring local and surrounding information. To mitigate domain shift and improve generalization, Aslani et al. (32) introduced a regularization network that incorporates an auxiliary loss term into a traditional encoder-decoder architecture.

Our approach outperformed some of the above-mentioned approaches. The effectiveness of DenseLesdoes not depend on the size of the lesions.

The vast majority of lesion segmentation methods operate on 3D images, allowing spatial information to be used in all directions. In our approach, we used information only from 2D slices, but we believe that it is not a disadvantage in training for DenseLes. We trained our models in three orthogonal directions (axial, coronal, and sagittal) and combined their information to produce the final segmentation result. With this step, we addressed the lack of 3D spatial information and filtered out numerous false objects. Furthermore, it makes the calculation less demanding and faster.

While 2D networks are often favored for their computational efficiency, they can face limitations in volumetric medical imaging, including a loss of inter-slice context and a greater susceptibility to “checkerboard” artifacts in anisotropic data. Furthermore, without sufficient volumetric information, very small or dispersed lesions can be difficult to distinguish from noise. Our proposed DenseLes approach effectively mitigates these challenges by utilizing dense convolutional connections and multi-scale feature extraction. This architecture ensures that, even within a 2D framework, the model maintains a high degree of feature reuse and spatial awareness, enabling it to identify dispersed lesions and preserve spatial continuity with high precision.

The error map indicates that the weaknesses of supervised and unsupervised methods are similar. We observed that segmenting small objects was more challenging for the reviewed approaches.

Performance was evaluated across small, medium, and large lesion strata. While DenseLes maintained high accuracy across all groups, we acknowledge that the limited number of medium- and large-sized lesions in the study population—consistent with typical MS lesion distribution—may limit the generalizability of the findings to these specific subgroups. Future studies using datasets with a higher prevalence of larger lesions will be necessary to further confirm these results.

A last contribution of our proposed pipeline is the automated anatomical classification of segmented lesions into distinct brain regions (e.g., periventricular, juxtacortical, and infratentorial). This feature provides clinicians with objective, region-specific metrics, such as lesion count and total lesion volume per location. This capability is clinically valuable, enabling the tracking of longitudinal changes in specific brain areas.

We noted that a separate, manually curated ground-truth dataset for anatomical localization was not available for this study. Therefore, the reliability of this classification is intrinsically dependent on the accuracy of the initial lesion segmentation. However, a formal, substantive validation of the classification component itself remains an important direction for future work.

The methodology of many approaches has been published. The source code for the proposed segmentation model, including training and evaluation scripts, is publicly available on GitHub at: https://github.com/szte-nirg-code/DenseLes.

In conclusion, the developed DenseLesalgorithm is extensively validated and is a fast, robust automated lesion segmentation that can also be applied to images from other MR vendors.

## Data Availability

The data analyzed in this study is subject to the following licenses/restrictions: the locally collected data are available upon request and may be released after approval by the Ethics Committee. Requests to access these datasets should be directed to office.radio@med.u-szeged.hu.

## Footnotes

1
**https://www.nitrc.org/projec/ts/msseg**

2
https://zenodo.org/records/10122614

3
https://fsl.fmrib.ox.ac.uk/fsl/fslwiki/BIANCA

4
https://www.nitrc.org/projects/flexconn

5
https://www.applied-statistics.de/lst.html
